# Minimally Invasive Surgery for Clinical Crown Lengthening Using Piezoelectric Ultrasound

**DOI:** 10.1155/2020/7234310

**Published:** 2020-02-29

**Authors:** Ana Carolina Monachini Marcantonio, Guilherme José Pimentel Lopes de Oliveira, Cássio Rocha Scardueli, Camila Chiérici Marcantonio, Rosemary Adriana Chiérici Marcantonio, Elcio Marcantonio

**Affiliations:** ^1^Department of Diagnosis and Surgery, Univ. Est. Paul. – UNESP, Araraquara 14801-903, Brazil; ^2^Department of Periodontology/Implantology, Dental School, Federal University of Uberlândia-UFU, Uberlândia 38405-266, Brazil

## Abstract

This case report is aimed at describing a flapless, minimally invasive clinical crown lengthening with an osteotomy performed using a piezoelectric ultrasound. A female patient complained about the amount of gum that was exposed when she smiled, which caused aesthetic discomfort. After a clinical examination, it was confirmed that the patient had excessive gum exposure in the upper arch of the dental region for teeth 14 to 24 when she smiled. The tomographic exam showed that bone tissue was at the level of the enamel-cementum junction, and gingival tissue covered a part of the anatomic crown. Virtual analysis using digital smile design (DSD) demonstrated that enlarging the clinical crowns would provide better aesthetics. The excess gingival tissue was removed from the gingival margin region with the aid of a mockup without interference to the interdental papillae. Then, osteotomy was performed using piezoelectric ultrasound until there was a 2.5 mm distance from the top of the bone crest to the new gingival margin. In the postoperative period, good repositioning of the gingival margin, absence of postoperative complications, and rapid healing of the gingival tissue were verified. After 6 months, a good aesthetic outcome was observed with stability in the level of the periodontal tissues obtained via the crown-lengthening technique. It can be concluded that the minimally invasive clinical crown-lengthening technique was effective in repositioning the gingival margin with no postoperative complications.

## 1. Introduction

The use of minimally invasive surgical techniques has been a trend in medicine and dentistry because they result in good clinical outcomes with minimal complications during the postoperative phase [1, 2]. Using minimally invasive surgical techniques has been related to reduced postoperative discomfort [3], reduced consumption of analgesic and anti-inflammatory drugs [4], reduced length of hospitalization, and, consequently, reduced time required to return to normal activities [4]. Thus, although the use of the new technologies may increase the cost of the surgical procedure, the patient's earlier return to normal life may generate benefits that makes the procedure more cost effective than if conventional surgical techniques are applied [5].

Specifically, in the dentistry field, efforts have been made to develop minimally invasive surgical techniques, such as the use of a surgical microscope [6], computed tomographic-guided surgeries [7], and the use of planning software, such as digital smile design (DSD), that assist in predicting the tissue to be excised for patient's oral rehabilitation [8]. The use of these technologies has promoted advances in minimally invasive techniques, such as the development of ultraconservative flaps with minimal detachment [9]. Minimally invasive surgical techniques have been indicated for the harvest of intraoral bone grafts [10], coverage of gingival recessions with subepithelial connective tissue grafts [6], treatment of periodontal bone defects [2], and immediate implant placement [11] with high levels of success and reduced patient morbidity.

One of the possible indications for the use of minimally invasive surgery is anterior crown lengthening indicated due to passive eruption disturbances. In this condition, the top of the bony crest is close to the cementum-enamel junction, and simply removing the gingival tissue is not sufficient to predictably expose the clinical crown over long follow-up periods [12, 13]. Indeed, a previous study described a minimally invasive technique for clinical crown lengthening with or without the aid of a microscope, showing that the flapless technique and osteotomy with a microchisel was as effective as the use of the open-flap technique promoting less side effects [12]. The osteotomy procedure can alternatively be performed with a piezoelectric ultrasound [14, 15]. This type of tool has the potential to remove the bone tissue without causing damage to the root surface and soft tissue and may be the most suitable for crown-lengthening surgical procedures that use a flapless technique during osteotomy.

Thus, the objective of this case report was to present a minimally invasive crown-lengthening technique with papilla preservation and an osteotomy performed by piezoelectric ultrasound.

## 2. Case Presentation

**Figure 1:**
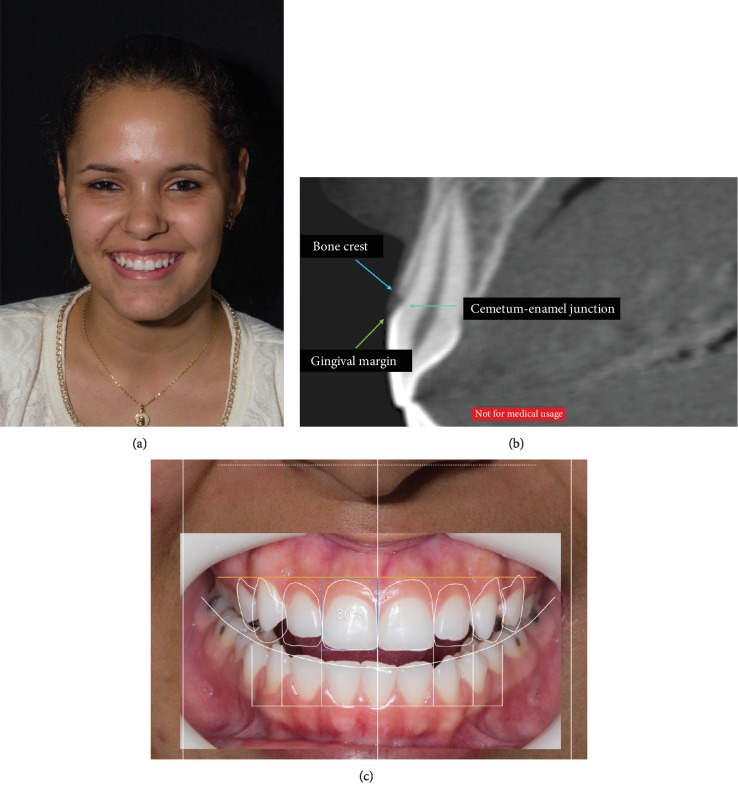
Initial diagnosis. (a) Initial condition of the patient where it is possible to observe a large gingival exposure when smiling. (b) Tomographic analysis showing that the top of the bone crest was practically at the level of the enamel-cementum junction. (c) DSD planning that showed the need to increase the length of the clinical crowns for better aesthetics.

A 22-year-old female patient complained about the amount of gingiva that was exposed when she smiled, which caused aesthetic discomfort for the patient. In the anamnesis, the patient reported not having any systemic problems, not continuously using any medication, and not being a smoker. A clinical examination confirmed the presence of an excessive exposure of the gingiva in the upper arch of teeth 14 to 24 (Figure 1(a)). The periodontal examination, which consisted of an analysis of gingival marginal bleeding, bleeding on probing, periodontal probing depth, and gingival margin position using a millimeter probe, confirmed that the periodontal tissues were in a healthy condition. In addition, conical beam tomography with an oral retractor was ordered to evaluate the bone and gingival tissue associated with the upper anterior teeth, and molding and photographs were also ordered.

The tomography showed that the bone tissue was at the level of the enamel-cementum junction and that the gingival tissue covered part of the anatomic crown (Figure 1(b)). Virtual planning was also performed using DSD, which showed the need to increase the size of the clinical crowns for better aesthetics (Figure 1(c)). With these data, it was shown that the patient suffered from altered passive eruption and, therefore, would need an osteotomy to enlarge the clinical crown. After this diagnosis, a flapless, minimally invasive surgical technique with piezoelectric ultrasound to perform the osteotomy and a mockup to guide the removal of soft tissue was proposed for the patient.

**Figure 2:**
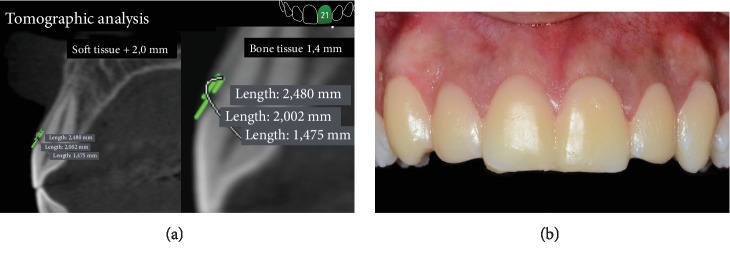
Planning the osteotomy and mockup. (a) Tomographic planning shows where the bone and gingival tissue should be removed. (b) Mockup in position that served as a guide for the removal of soft tissue from the gingival margin.

Surgical planning was performed by DSD with the patient's smile photos and crown measurements using an 80% width to length ratio. After design of the optimal teeth, the distance from the cementum-enamel junction to the top of the bone crest and the position of the gingival margin in relation to the enamel-cementum junction was measured by tomographic analysis (Figure 2(a)). These measures were used as a reference for the wax model and mockup (Figure 2(b)). In addition, these measurements were used with tomography to plan the amount of osteotomy needed to maintain the biological space without recurrence of coronary covering by the gingival tissue.

**Figure 3:**
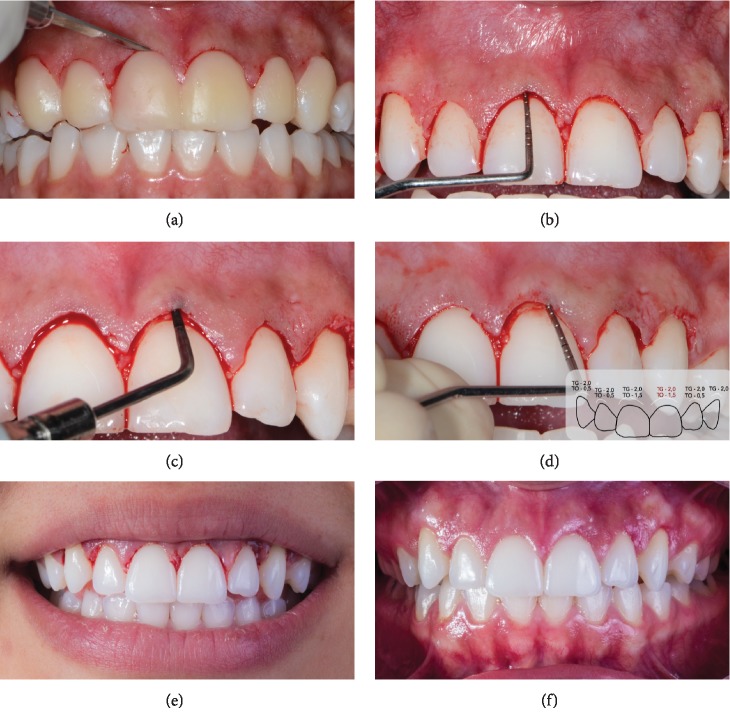
Surgical procedure. (a) Initial incision with the aid of a mockup that was used as a guide for gingival tissue removal. (b) During the probing procedure, it was verified that the distance from the gingival margin to the top of the bone crest was 1 mm, which meant that the osteotomy procedure was necessary. (c) Osteotomy was performed with piezoelectric ultrasound without flap elevation and without interference in the interdental papillae. (d) The procedure removed 1 mm of bone tissue to restore the biological space, taking into account the thin gingival phenotype presented in the patient. (e) Immediate postoperative situation where it is possible to perceive a minimally traumatic clinical condition. (f) The good postoperative condition observed 6 months after the surgical procedure: the periodontal tissue was completely healed.

With the mockup in position, the projection of what the gingival condition would look like after the surgical procedure was shown to the patient. After patient approval, the clinical crown-lengthening procedure was performed; this procedure consisted of marking the soft tissue height to be removed (Figure 3(a)) and a sulcular incision to allow the removal of a gingival collar at the buccal face around all anterior and upper teeth without interfering with the interproximal region.

After removing the gingival collar, the need for bone tissue removal due to the proximity of the new gingival margin was detected, since the bone tissue was practically at the level of the enamel-cementum junction (Figure 3(b)). A flapless osteotomy procedure was performed at the buccal bone with the aid of a piezoelectric ultrasound that induces bone tissue wear by ultrasonic vibrations (Figure 3(c)). According to the treatment plan, approximately 2.5 mm of bone was removed around all of the upper anterior teeth. Osteotomy was performed with minimal invasiveness, cutting only the bone tissue without causing damage to the root surface (Figure 3(d)).

**Figure 4:**
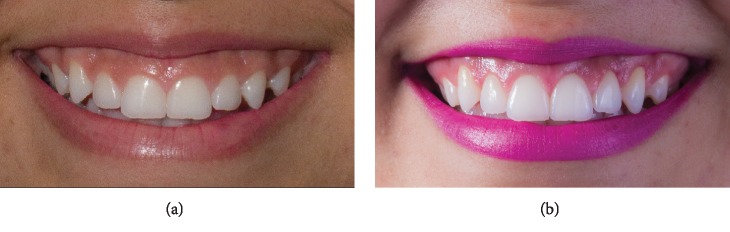
Clinical condition. (a) Before the surgical procedure. (b) Fourteen days after the surgical procedure. It is observed that the applied surgical technique promoted adequate increase in the clinical crown of the teeth and rapid recovery of periodontal tissues during the postoperative phase.

Then, the osteotomy height was confirmed by probing (Figure 3(d)), with the immediate results demonstrating an increase in the clinical crown length with minimal soft tissue trauma (Figure 3(e)). After 6 months, a good aesthetic result was observed with stability in the results obtained in the clinical crown-lengthening technique (Figure 3(f)).  Figure 4 shows the initial condition (Figure 4(a)) and final clinical condition 14 days after the surgical procedure (Figure 4(b)).

## 3. Discussion

This case report shows a flapless, minimally invasive clinical crown-lengthening technique and osteotomy performed with piezoelectric ultrasound with a good clinical outcome; it was possible to correct the altered passive eruption with no complications in the postoperative period.

Altered passive eruptions are one of the main indications for clinical crown-lengthening procedures [12]. In these cases, the margin of the bone tissue does not normally migrate apically after the establishment of dental occlusion, which results in an excessive covering of the clinical crown [13] and causes discomfort to the patients regarding the aesthetics of the upper teeth. Usually, the treatment of this type of condition is indicated in cases where there is presence of thick gingival phenotypes associated with a keratinized gingival width higher than 2 mm [12]; however, the occurrence of root exposure or return of the gingival margin in an excessive coronal position after the treatment are possible findings that should be carefully evaluated during the follow-up visits.

The conventional surgical procedure in these cases would be to remove excess gingival margin tissue with a flap detachment and osteotomy until the establishment of biological distances; however, because it is sometimes necessary to open the flaps and because the osteotomy is a more intense dental procedure, undesirable side effects, such as bleeding, inflammation, and postoperative pain, are expected [12, 13]. Thus, using a minimally invasive procedure for this type of surgical technique could have great benefits for the postoperative recovery of these patients.

The surgical technique applied in this study was based on DSD planning that allowed the production of a mockup that guided the amount of soft tissue to be removed in the primary incision [8]. For this aim, it was necessary to obtain a CT scan that allowed planning for the amount of soft tissue and bone tissue that should be removed during the surgical procedure. In addition, through the initial photographs, the original size of the teeth was predicted to assess the golden proportions [8]. The use of DSD allowed minimal removal of the soft tissue at the gingival margins without the interference of the interproximal tissue, keeping the flap closed throughout the surgical procedure.

Another important technical detail of the surgical technique was that the osteotomy procedure was performed with piezoelectric ultrasound. Hand and rotary instruments are traditionally used to perform osteotomies [10, 14, 16]; however, the flapless technique causes difficulties in the use of these traditional instruments. Rotary instruments for osteotomy procedures have been the treatment of choice in different clinical applications; however, the use of these instruments has been related to thermal damage that may alter bone tissue stability after the surgical procedure [17]. Furthermore, the use of rotatory instruments with a flapless technique may induce lesions in the soft tissues and on the root surfaces [12]. A piezoelectric ultrasound has been shown to have good efficacy in the osteotomy procedure with minimal thermal damage and reduced possibility of soft tissue and root surface injury [3, 10]. In addition, it has also been shown that sites where an osteotomy was performed with piezoelectric ultrasound have lower expressions of biological mediators of osteoclastogenesis and inflammation such as RANKL [15] and IL-1*β* [3], which may allow better postoperative outcomes for the patient. A limitation regarding the use of the piezoelectric ultrasound is that there is a longer clinical time required for the osteotomy procedure than the time required for osteotomies performed with rotary instruments [10, 18]; however, since the use of piezoelectric ultrasound allows for osteotomies with a flapless technique, the suture procedure is not necessary and the surgical time may be compensated for when compared to rotary instruments.

## 4. Conclusion

It can be concluded that using a minimally invasive clinical crown-lengthening technique was effective in repositioning the gingival margin when used with an osteotomy and presented no postoperative complications. However, randomized controlled clinical trials, with longer periods of follow-up, should be conducted to compare the effectiveness of this minimally invasive technique compared to those traditionally used for clinical crown-lengthening procedures in altered passive eruption disorders.
